# Reliability, validity, and usability of an 18-item immersive virtual reality action research arm test: a validation study in stroke survivors

**DOI:** 10.3389/fneur.2026.1876892

**Published:** 2026-07-22

**Authors:** Zhipeng Pan, Hewei Wang, Yue Sun, Zhen Wei, Hongrui Liu, Jingyi Wei, Cui Yu, Xinchun Dong, Jianxia Lu, Bin Su

**Affiliations:** 1 School of Rehabilitation, Shandong University of Traditional Chinese Medicine, Jinan, China; 2 Department of Rehabilitation, Huashan Hospital, Fudan University, Shanghai, China; 3 School of Exercise and Health, Nanjing Sport Institute, Nanjing, China; 4 School of Rehabilitation, Jiangsu Medical College, Yancheng, China

**Keywords:** ARAT, hand tracking, reliability, stroke, upper extremity, validity, virtual reality

## Abstract

**Background:**

Stroke is a leading cause of adult disability, with upper extremity hemiparesis affecting approximately 80% of survivors. While the Action Research Arm Test (ARAT) is the reference standard for assessing motor function, its clinical use is hindered by being resource-intensive and requiring standardized physical props. Virtual Reality (VR) has emerged as a promising method for automated and objective assessment.

**Objective:**

This study aimed to develop and validate an 18-item immersive VR version of the ARAT (ARAT-VR) and assess its validity, reliability, and usability among stroke survivors, healthcare professionals, and healthy controls.

**Methods:**

Thirty stroke survivors, 30 healthy controls, and eight healthcare professionals were recruited. The ARAT-VR system, developed for the PICO 4 Ultra Enterprise headset, utilizes an iToF depth sensor for controller-free optical hand tracking and retains 18 of the original 19 items. Content validity was evaluated by professionals. Concurrent validity was measured via Spearman's correlation between ARAT-VR and traditional ARAT scores. Test-retest reliability (Intraclass Correlation Coefficient, ICC) was assessed over a 7-day interval. Usability was measured using the System Usability Scale (SUS).

**Results:**

Regarding content validity, most of the 18 ARAT-VR items were rated as comparable to physical tasks specifically in visual-motor control demands (Median = 0), except for manipulating minute objects (e.g., 6 mm marbles), which was rated as more difficult (1.0 [1.0–1.0]). Regarding concurrent validity, the ARAT-VR scores for the paretic hand strongly correlated with both the 18-item traditional ARAT (*r*_s_ = 0.845, 95% CI: 0.673–0.965, *p* < 0.001) and the full 19-item ARAT (*r*_s_ = 0.845, 95% CI: 0.696–0.961, *p* < 0.001). Bland-Altman analysis indicated a mean bias of 1.77 points with 95% limits of agreement from −10.07 to 13.60. Test-retest reliability was excellent (ICC = 0.98, 95% CI: 0.96–0.99, *p* < 0.001), with a Minimal Detectable Change (MDC) of 5.36 points, representing the minimum change required to exceed measurement error. Usability was rated as excellent (Median SUS = 85.0 for healthy controls and 81.3 for stroke survivors; *p* = 0.212).

**Conclusions:**

Initial findings suggest that the 18-item ARAT-VR possesses favorable concurrent validity, high test-retest reliability, and acceptable usability, highlighting its potential as a supplementary digital assessment tool for stroke survivors. The calculated MDC of 5.36 points offers a preliminary reference for future longitudinal monitoring. However, its readiness for routine clinical implementation remains unestablished. Further large-scale studies are necessary to validate the system across broader populations and explore hardware optimizations, such as the integration of haptic feedback.

## Introduction

As a leading cause of mortality and long-term disability worldwide, stroke places an immense strain on both societal and healthcare resources (1–3). Upper extremity (UE) hemiparesis is a particularly common sequela, affecting roughly 80% of individuals post-stroke (2, 4). These motor deficits, characterized by reduced strength, coordination, and manual dexterity, often result in significant activity limitations that severely compromise the stroke survivor's independence and quality of life (5).

To maximize therapeutic efficacy, it is essential to perform precise, frequent, and objective evaluations of upper extremity function. The International Classification of Functioning, Disability and Health (ICF) framework suggests that such assessments should span body function, activity, and participation domains (6). Systematic monitoring not only tracks recovery trajectories but also equips clinicians with essential data to forecast outcomes and customize intervention plans (7, 8).

In the realm of clinical research and practice, the Action Research Arm Test (ARAT) is widely recognized as the reference standard for assessing upper extremity motor function due to its hierarchical structure, which comprehensively evaluates grasp, grip, pinch, and gross movements—domains that are critical for executing activities of daily living (ADLs) (9, 10). While it demonstrates robust psychometric qualities, such as high validity, reliability, and responsiveness (11–13), traditional administration is resource-heavy. It requires specific physical props and the constant supervision of a trained clinician for manual scoring. Such logistical barriers restrict assessment frequency in high-volume clinical environments and impede the adoption of tele-rehabilitation or remote monitoring strategies (14, 15). While clinically valuable, the conventional ARAT requires standardized physical props, dedicated clinical space, and manual administration. These requirements can make frequent serial assessments time-consuming and susceptible to inter-rater variability. Consequently, there is a need for valid and automated assessment alternatives. Beyond establishing statistical equivalence, the ARAT-VR aims to address these logistical limitations. By digitizing physical props and automating the scoring workflow, the VR system reduces equipment dependencies and may decrease both clinician burden and scoring subjectivity. Furthermore, optical tracking within VR platforms can capture kinematic metrics (e.g., movement trajectory and velocity) without external sensors, offering supplementary insights into movement quality and compensatory strategies.

Virtual Reality (VR) has gained traction in neurorehabilitation as a powerful modality, providing immersive settings that enhance neuroplasticity and patient engagement (16–18). Recent studies have indicated that adjunctive VR-based rehabilitation can enhance upper limb motor recovery across multiple functional domains compared to conventional occupational therapy alone. Interestingly, these studies highlight a technological dichotomy: while fully immersive VR setups are highly effective for gross motor function, non-immersive platforms have historically been more conducive to refining fine motor dexterity (19–21). Beyond its therapeutic role, VR holds considerable promise as an automated evaluation instrument. By utilizing optical tracking and built-in sensors, VR platforms can generate quantitative, objective kinematic metrics, such as movement trajectory, smoothness, and velocity. If properly extracted and analyzed, these objective data could provide clinicians with additional contextual information to supplement traditional ordinal scoring, potentially aiding in the observation of movement quality and compensatory patterns (22–24).

However, before integrating such kinematic analyses into clinical routine, it is first necessary to establish that virtual assessment environments can reliably replicate the foundational psychometric properties of established physical tests. Numerous studies have successfully validated the feasibility and efficacy of this technological transition. For instance, in the assessment of gross manual dexterity, several researchers have developed virtual versions of the Box and Blocks Test (VR-BBT) utilizing either hand-tracking or controller-based methods (25–27). Although these studies consistently observed that operation speeds in virtual environments were slower than in physical settings due to the lack of tactile feedback (TF), the VR versions demonstrated high correlation and test-retest reliability with the traditional BBT, confirming their validity as assessment tools for people with stroke and Parkinson's disease. Similarly, the recent development of an immersive 6-min Pegboard and Ring Test (6PBRT-VR) by Maden et al. (28) exhibited excellent test-retest reliability and concurrent validity in healthy young adults, further expanding the potential of VR in assessing upper extremity endurance and fine motor function.

Nevertheless, translating the full complexity of the ARAT into a virtual environment poses technical challenges, particularly regarding fine motor skills. For instance, Burton et al. (29) previously designed an immersive ARAT-VR system but were forced to exclude six of the 19 items (e.g., manipulating a 0.6 cm marble) and relax scoring criteria due to occlusion issues and hand-tracking inaccuracies at the time. While statistically valid, this abridged version offered limited insight into fine pinch function, potentially failing to capture the full spectrum of hand function recovery in clinical populations.

Building on previous adaptations, this study developed an immersive, kinematic-based screening tool adapted from the ARAT. We utilized the PICO 4 Ultra Enterprise headset, equipped with a Snapdragon XR2 Gen 2 chipset. By leveraging its integrated iToF (indirect Time-of-Flight) depth sensor and high-resolution RGB cameras for robust spatial tracking, this system facilitated the controller-free implementation of 18 ARAT items (30). This technological upgrade allowed us to adapt 18 of the original 19 ARAT items (excluding only the “hand behind head” task due to occlusion) into a prop-free virtual environment. By automating standard temporal scoring rules, this system is designed to complement existing clinical assessments by evaluating motor control and spatial trajectories without the need for physical props.

The primary aim of this study was to develop this 18-item ARAT-VR and validate it among healthy controls and stroke survivors. We sought to establish concurrent validity, hypothesizing a strong correlation between ARAT-VR and traditional ARAT scores, especially for the paretic hand. Furthermore, we hypothesized that high-functioning individuals (healthy controls and stroke survivors using their non-paretic hand) would attain comparably high scores in both formats, indicating minimal ceiling effects. Secondary goals included evaluating the system's usability and test-retest reliability.

## Methods

### Study design and participants

This observational validation study, incorporating both cross-sectional and short-term test–retest reliability components, included stroke survivors, healthy controls, and healthcare professionals. Participants were recruited from the Yancheng NO.1 People's Hospital between August 2025 and January 2026. To minimize selection bias, a consecutive sampling approach was employed. All individuals with stroke admitted to the rehabilitation ward with a primary diagnosis of stroke during the study period were prospectively screened for eligibility. Written informed consent was obtained from all participants. This manuscript is reported in accordance with the STROBE statement for observational studies. Measurement properties were described following COSMIN terminology, including criterion-related validity, test–retest reliability, and measurement error (Supplementary Table 1).

**Figure 1:**
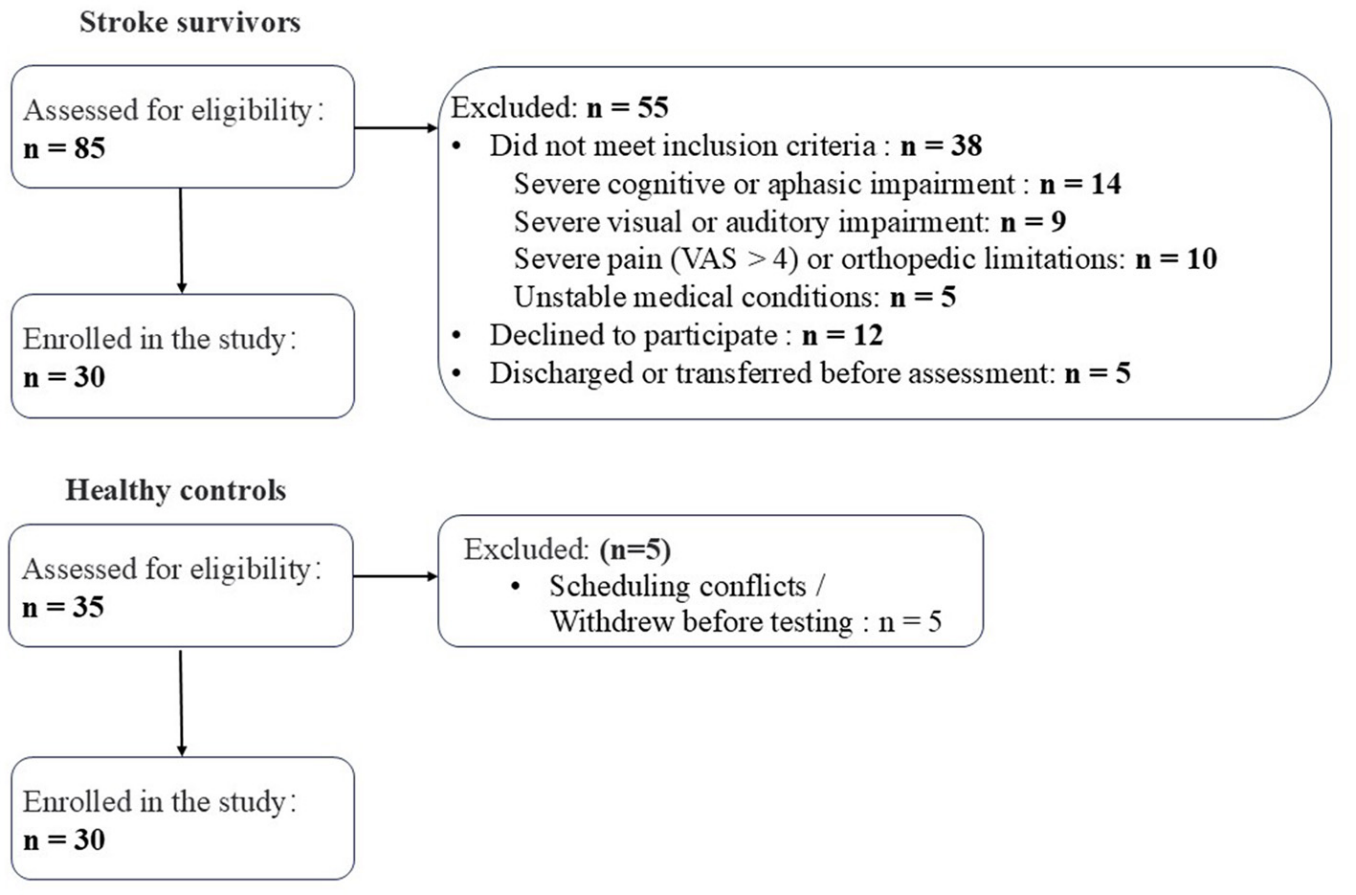
Participant flow diagram.

As detailed in the Participant Flow Diagram (Figure 1), a total of 85 stroke survivors were initially assessed. Of these, 55 were excluded: 38 did not meet the clinical inclusion criteria (e.g., severe cognitive or aphasic impairments preventing instruction comprehension, significant visual deficits, or severe shoulder pain/orthopedic limitations), 12 declined to participate, and five were discharged or transferred prior to assessment. Consequently, a final cohort of 30 stroke survivors was enrolled.

Concurrently, 35 healthy volunteers were screened via community advertisements and staff networks. Five were excluded (due to scheduling conflicts). A final cohort of 30 healthy control subjects (HCS) was enrolled. The healthy cohort was specifically included to establish normative baseline usability, identify potential ceiling effects inherent to the VR technology, and evaluate the discriminant validity of the ARAT-VR system.

During screening, all enrolled participants self-reported no prior experience with immersive VR or VR-based gaming systems.

### Inclusion criteria

People with stroke must (1) meet the diagnostic criteria for stroke, confirmed by computed tomography (CT) or magnetic resonance imaging (MRI) to have hemorrhage or ischemic stroke, (2) have stable condition, no severe cognitive impairment (MoCA ≥ 23), and ability to cooperate with the therapist to complete the trial (defined as the capacity to follow 2-step verbal commands and maintain independent sitting balance), (3) have intact visual field or normal after correction, (4) voluntarily participate in the study while agreeing to sign the informed consent form.

### Exclusion criteria

People with stroke will be excluded if they (1) have a history of traumatic brain injury, Parkinson's disease, psychiatric disorders, drug abuse, or alcohol abuse, (2) have severe comprehensive aphasia making it difficult to follow the therapist's instructions, (3) have severe hearing loss, (4) have hemispatial neglect or visual impairment, (5) have severe sensory dysfunction in the hemiplegic upper limb, (6) have obvious pain (rated >4 on the Visual Analog Scale) or orthopedic limitations (e.g., severe shoulder subluxation, shoulder-hand syndrome) in the paretic upper limb, which could restrict active range of motion and confound motor capability with pain avoidance, (7) have obvious VR motion sickness unable to tolerate the trial, (8) have vital organ failure or other critical illnesses, or (9) have not signed the informed consent form.

### Healthy controls

Participants must (1) have no history of major physical or mental illnesses and no history of long-term use of medications affecting the central nervous system, (2) have no cognitive impairment, (3) have no obvious visual or hearing impairments (after correction) or osteoarticular diseases affecting upper limb activities, (4) have comparable demographic data such as age and handedness with the stroke survivor group, and (5) be fully informed and voluntarily sign the informed consent form. Healthcare professionals must have more than 3 years of experience in rehabilitation practice.

## Materials

### The ARAT

**Figure 2:**
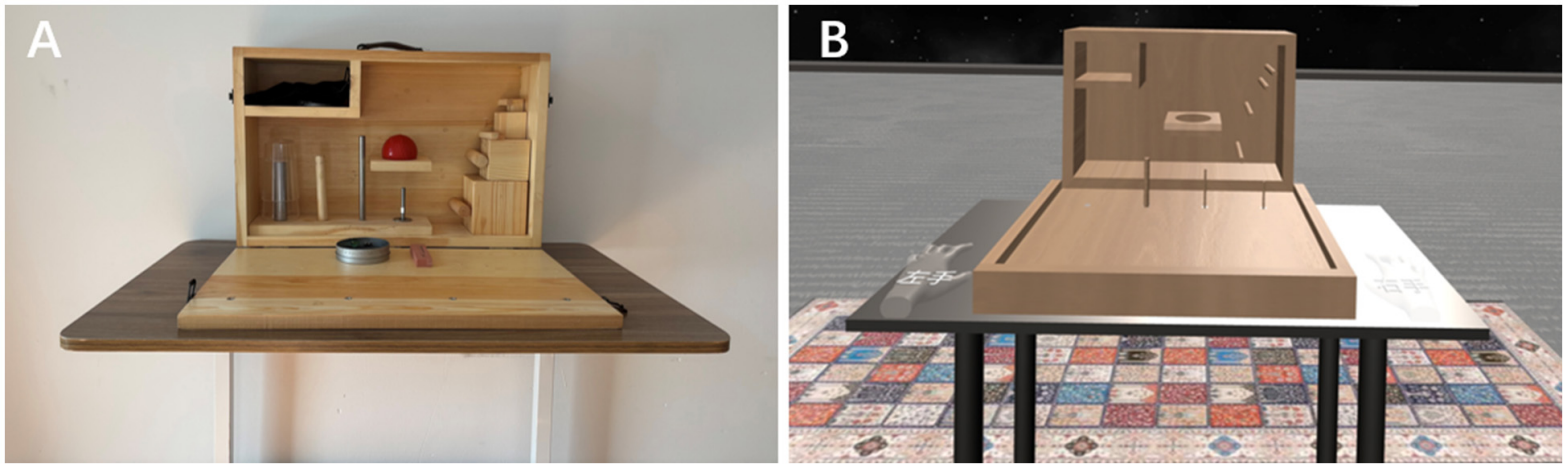
Comparison of the physical and virtual assessment environments. **(A)** The standardized setup of the traditional ARAT, featuring the physical test kit and workspace. **(B)** The ARAT-VR environment as seen through the headset. The virtual desk and objects were modeled at a 1:1 scale to match the physical dimensions.

The traditional ARAT (Figure 2A) was employed as the reference standard for assessing upper extremity motor function, serving as the benchmark for direct comparison and validation of the ARAT-VR system. This standardized measure consists of 19 items grouped into four subscales: Grasp (6 items), Grip (4 items), Pinch (6 items), and Gross Movement (3 items) (31). The Grasp subscale assesses the ability to handle objects of varying size and weight, requiring participants to lift wooden blocks (2.5 cm, 5 cm, 7.5 cm, and 10 cm), a cricket ball, and a sharpening stone onto a shelf. The Grip subscale evaluates hand dexterity and strength through tasks such as pouring water between glasses, displacing alloy tubes (2.25 cm and 1 cm diameter), and placing a washer over a bolt. The Pinch subscale focuses on fine manual dexterity, requiring the manipulation of marbles (1.5 cm and 6 mm diameter) using specific finger combinations (thumb with index, middle, or ring finger). Finally, the Gross Movement subscale assesses proximal upper limb function by asking participants to place their hand behind their head, on top of their head, and to their mouth. Each item is scored on a four-point ordinal scale ranging from 0 to 3 based on the quality and time of execution: three points for completing the task within 5 s with correct posture; two points for completing the task between 5 and 60 s; one point for partial completion where shoulder or elbow flexion was observed but the hand failed to reach the target within 60 s; and 0 points for no movement initiated within 60 s. The maximum total score is 57. Standard clinical administration typically utilizes Guttman scaling (32). Participants were required to perform all 19 items of the traditional physical ARAT. However, to ensure structural equivalence with the 18-item ARAT-VR, the total score for the traditional assessment was retrospectively recalculated based only on the 18 shared items (maximum score 54) for all comparative statistical analyses.

### The ARAT-VR system

**Figure 3:**
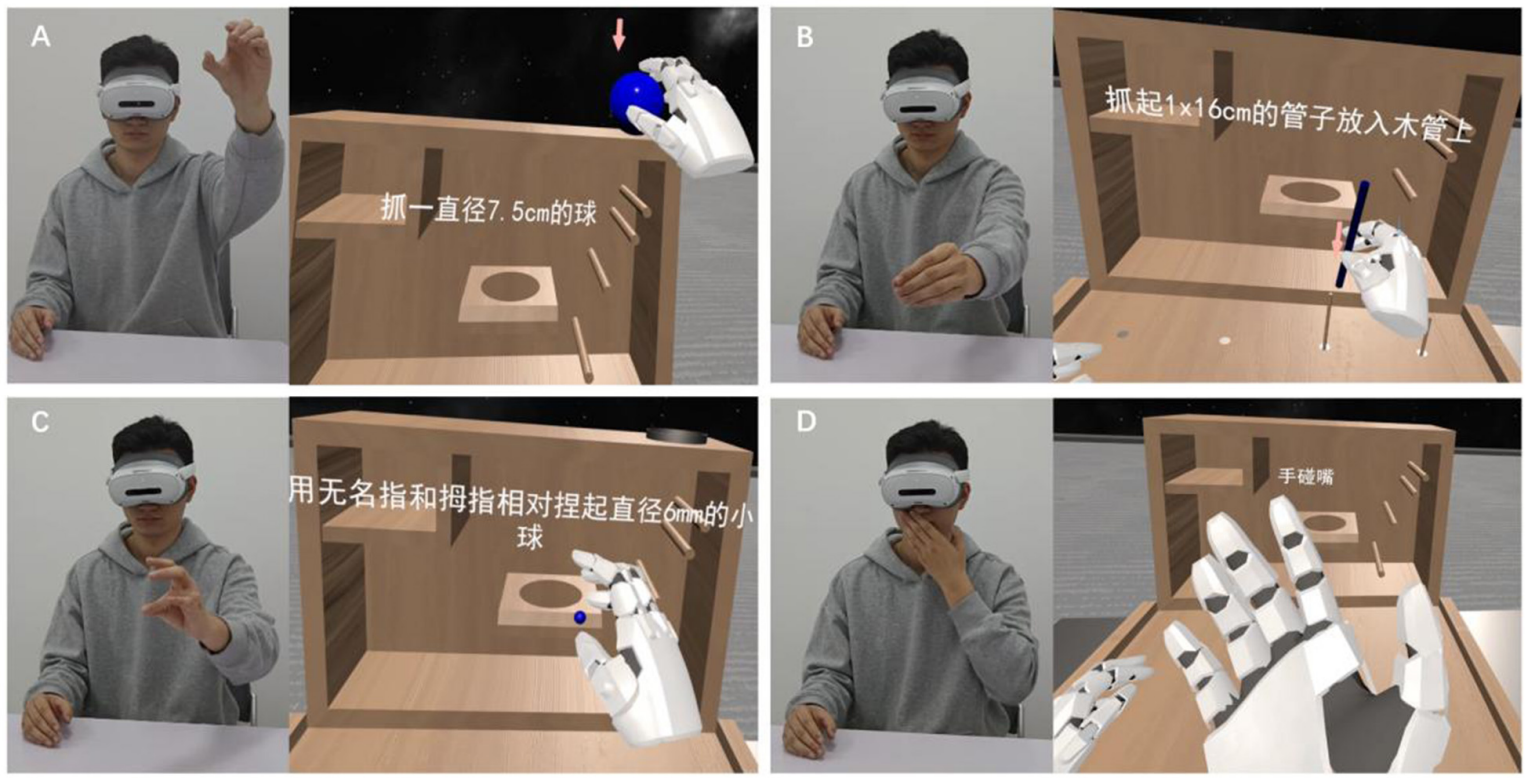
Representative tasks in the ARAT-VR assessment. The figure illustrates participants performing specific motor tasks (left panels) alongside the corresponding first-person view in VR (right panels). **(A)** Grasp Subscale: grasping and transporting a virtual 7.5 cm cricket ball to a target platform. **(B)** Grip Subscale: gripping a 1 cm × 16 cm alloy tube and placing it over a wooden peg. **(C)** Pinch Subscale: pinching and transporting a virtual 6 mm marble to a target platform using the thumb and ring finger. **(D)** Gross Movement Subscale: touching the mouth with the tested hand.

The ARAT-VR system was developed in C# language using the Unity 3D engine and executed on the PICO 4 Ultra Enterprise headset. This device utilizes the Snapdragon^®^ XR2 Gen 2 platform to ensure high-fidelity physics simulation and low latency. Optically, it features Pancake lenses with motorized interpupillary distance adjustment (58–72 mm) to optimize visual clarity for diverse users. Crucially, the tracking system integrates 32MP color passthrough and iToF depth sensing. This multi-sensor fusion provides the enhanced tracking stability required for robust optical hand-tracking during fine motor tasks (e.g., pinching small objects). The virtual environment was designed as a distraction-free room. To maximize ecological validity, the virtual testing apparatus was rigorously modeled as a high-fidelity digital replica of the standard physical ARAT kit (Figure 2B). The dimensions of the virtual desk and all objects were modeled at a 1:1 scale, ensuring that the spatial constraints and visual cues in VR strictly adhered to the physical standards used in clinical routine. To handle interactions with complex objects (e.g., cricket ball, sharpening stone) without visual clipping during spastic movements, the system employed Proxy Colliders attached to the metacarpophalangeal and interphalangeal joints. This was coupled with Continuous Collision Detection (CCD) to ensure stable rigid-body physics interactions, particularly for minute items like the 6 mm marble. Furthermore, the system incorporates OpenCV-based computer vision algorithms and predictive skeletal modeling to enable precise tracking of the 26 hand key points. In instances of severe self-occlusion during fine pinch tasks, the system utilizes physics-based Grab Anchors and trajectory extrapolation to maintain the spatial relationship between the fingers and the object. By addressing prior tracking constraints related to finger occlusion and collision instability, the system implemented 18 of the original 19 ARAT items (excluding only the “hand behind head” task) and strictly adheres to standardized protocols, enabling fully automated judgment and real-time scoring. Real-time clinical supervision was facilitated by wirelessly screen casting the user's view to an external tablet. This allowed therapists to monitor performance, remotely control the session flow (e.g., start, pause, or reset tasks), and ensure safety. Throughout the VR assessment, participants were continuously monitored by the clinical assessor for any signs of VR-induced adverse events, including cybersickness, dizziness, visual discomfort, or fatigue. Participants were instructed to verbally report any discomfort immediately, with the protocol allowing for immediate pausing or termination of the session if requested. Upon session completion, assessment data including item scores and execution times were automatically synchronized and stored on the external monitoring tablet. This setup facilitated immediate data review and ensured portability for clinical use. However, the manipulable objects remained purely virtual; the system did not simulate physical weight, load, or tactile resistance. Therefore, task completion primarily reflects kinematic execution and visuomotor control rather than force modulation. More details on the task execution and user interaction are provided in Figure 3 and in the supplementary movie file (Supplementary Table 1).

### Procedure

All assessments were conducted in a standardized clinical environment by separate assessors according to the blinding protocol described below. Participants were seated on a standard armless chair with back support, positioned in front of a height-adjustable table. For the ARAT-VR setup, the examiner fitted the headset on the participant and adjusted the interpupillary distance to ensure visual clarity. Prior to testing, a spatial calibration procedure was performed to align the position and height of the virtual desk with the physical table, ensure visuo-tactile colocation for the workspace surface. This provided a physical resting platform for the users' arms; however, the manipulable objects (e.g., marbles, blocks) remained purely virtual without physical counterparts. To ensure safety, the participant's field of view was wirelessly screencast to an external tablet, allowing the therapist to monitor task execution in real-time without physical interference. To minimize order and fatigue effects, a randomized order of administration was employed on Day 1, where participants completed both traditional ARAT and ARAT-VR assessments in a randomized order separated by a 15-min rest. Before the formal VR assessment, a standardized 3-min tutorial was administered to familiarize participants with gesture interactions. To evaluate test-retest reliability, participants returned for a second session after a 7-day interval to perform the ARAT-VR assessment only.

The trial was initiated remotely by the therapist via the tablet interface. Upon activation, the system presented a 1-min audiovisual tutorial, combining voiceover instructions with on-screen text to explain the task requirements and interaction mechanics. The formal assessment started automatically upon completion of the instructional tutorial. The system automatically detected movement onset. Crucially, to ensure the assessment functioned as an objective clinical tool, algorithmic compensation for patient motor deficits was explicitly omitted. Kinematic smoothing filters, grasp-release hysteresis loops, and time-to-grab confirmation thresholds (such as delays exceeding 300 ms) were not implemented in the tracking software. Additionally, target assistance mechanisms were disabled. This approach was adopted to ensure that the system recorded the stroke survivor's raw motor trajectories, including inherent tremors and spasticity, thereby maximizing the ecological validity of the evaluation. To guide user attention, target objects appeared with a highlighted edge upon spawning. The system provided immediate audiovisual feedback; the grasped object shifted color to blue accompanied by a synchronized simulated sound effect, thereby compensating to some extent for the lack of tactile feedback (TF). Subjective judgment was replaced by a fully automated scoring algorithm. The software precisely recorded execution times and assigned scores adhering to standardized ARAT thresholds.

An assessor blinding protocol was implemented to mitigate potential observer bias. The physical ARAT was administered and scored by an independent, experienced occupational therapist. A separate researcher, who was blinded to the participants' physical ARAT scores, supervised the ARAT-VR sessions to manage hardware setup and monitor safety. Furthermore, since the ARAT-VR utilizes a fully automated scoring algorithm, subjective rater bias was inherently controlled for in the virtual assessment arm. Notably, participant blinding was not feasible due to the overt nature of the head-mounted VR intervention.

Following the completion of both testing versions on Day 1, eight healthcare professionals assessed the content validity of the system using a comprehensive questionnaire. First, they rated the comparative difficulty of each of the 18 VR items against the ARAT on a five-point Likert scale (ranging from −2 “Much Easier” to +2 “Much Harder”) (33). Subsequently, they evaluated the system's ergonomic and technical quality across four dimensions, visual perception, hand tracking stability, interactive feedback, and overall ergonomics, using a five-point scale (1 = Very Poor to 5 = Excellent). Additionally, to evaluate the system's ease of use and acceptance, all participants (stroke survivors and healthy controls) completed the System Usability Scale (SUS) (34) immediately after their first VR session.

### Statistical analyses

All statistical analyses were performed using de-identified datasets by an independent analyst who was not involved in participant assessments. Statistical analyses were performed using IBM SPSS Statistics 27.0, and data visualization was conducted with GraphPad Prism 10.1, with the significance level set at α = 0.05 *A priori* sample size estimation using G^*^Power 3.1 indicated that a minimum of 30 participants was required to detect a correlation coefficient of *r* ≥ 0.50 (power = 0.80, α = 0.05). However, this sample size calculation was explicitly powered for correlation analysis, not for ensuring precise Bland-Altman limits of agreement or narrow ICC confidence intervals. Consequently, this represents a preliminary psychometric validation.

Although individual items of the ARAT and ARAT-VR are ordinal data, the total scores derived from summing these items provide a broader range of values and are theoretically treated as approximating continuous (interval) data. Given the moderate sample size (*N* = 30), distribution normality was evaluated using the Shapiro-Wilk test. As the majority of variables exhibited non-normal distributions (*p* < 0.05 in most cases), non-parametric tests were employed for inferential analyses. Descriptive statistics are reported as median (interquartile range [IQR]) for ordinal and non-normally distributed data, and as mean ± standard deviation (SD) for normally distributed continuous variables.

Content validity was presented as Median (Interquartile Range [Q1–Q3]) based on a five-point Likert scale (−2 = much easier in VR; 0 = equivalent; +2 = much harder in VR) for each item to evaluate difficulty equivalence between VR and physical tasks. Concurrent validity was evaluated by computing Spearman's rank correlation coefficients (*r*_s_) between the ARAT-VR scores (Day 1) and both the adjusted traditional ARAT (18 items, excluding the “hand behind head” task) and the full traditional ARAT (19 items). Correlations were classified as negligible (0.00–0.30), low (0.30–0.50), moderate (0.50–0.70), high (0.70–0.90), or very high (0.90–1.00) (35). Agreement was assessed with Bland-Altman plots based on the 18 shared items to detect systematic bias. Discriminant validity was tested using the Mann–Whitney *U*-test, comparing ARAT-VR scores for the paretic hand in stroke survivors vs. the non-dominant hand in healthy controls.

Test-retest reliability was evaluated with the Intraclass Correlation Coefficient (ICC, model 3, 1; two-way mixed effects, absolute agreement) between Day1 and Day7 VR sessions. ICC values were interpreted as poor (< 0.50), moderate (0.50–0.75), good (0.75–0.90), or excellent (>0.90) (36). Measurement error was determined by the Standard Error of Measurement (SEM = SD × √ (1–ICC)) and the Minimal Detectable Change at 95% confidence (MDC95 = 1.96 × SEM × √2). Learning effects were assessed with the Wilcoxon signed-rank test for differences between Day 1 and Day 7 scores.

System Usability Scale (SUS) scores were summarized descriptively. Total SUS scores were interpreted using standard percentiles, with scores >68 indicating “Above Average” and >80 indicating “Excellent” usability.

## Results

Eight healthcare professionals took part in the content validity phase of the trial. Among these, five were occupational therapists and three were physical therapists. All professionals possessed more than 3 years of clinical experience and were familiar with the ARAT prior to the experiment.

Thirty healthy control subjects (19 males/11 females) with a mean age of 56.9 ± 5.7 years, and 30 individuals with stroke (20 males/10 females) with a mean age of 58.8 ± 8.5 years participated in the study. Individuals with stroke presented with a mean time since stroke onset of 5.0 ± 3.8 months. The cohort included 21 ischemic and nine hemorrhagic cases, with 17 presenting with right-sided and 13 with left-sided hemiparesis. Detailed demographic and clinical characteristics are presented in Table 1.

**Table 1:** Demographic and clinical characteristics of participants.

Characteristics	Stroke survivors (*n* = 30)	Healthy controls (*n* = 30)	*p*-value
Age (years), Mean ± SD	58.8 ± 8.5	56.9 ± 5.7	0.321
Gender (Male/Female), *n*	20/10	19/11	0.787
Handedness (Right/Left), *n*	30/0	28/2	0.492
Education (years), Mean ± SD	9.5 ± 3.1	10.1 ± 2.9	0.441
Disease Duration (months), Mean ± SD	5.0 ± 3.8	—	—
*Chronicity (< 1*/*1–6*/*>6 months), n*	5/20/5	—	—
Stroke type (Ischemic/Hemorrhagic), *n*	21/9	—	—
Paretic side (Right/Left), *n*	17/13	—	—
Brunnstrom stage (I–III/IV/V/VI), *n*			—
Upper Limb	4/5/15/6	—	—
Hand	5/2/14/9	—	—
MoCA Score (/30), Mean ± SD	25.2 ± 1.3	—	—
FMA-UE Score (/66), Mean ± SD	45.4 ± 16.1	—	—
*Severity (Severe*/*Moderate*/*Mild*)^a^, *n*	5/10/15	—	—
ARAT Score (/57), Median (IQR)			
Affected/Non-dominant Hand	38.5 (28.0–49.8)	57.0 (57.0–57.0)	—
Less-affected/Dominant Hand	57.0 (56.0–57.0)	57.0 (57.0–57.0)	—

Values are presented as mean ± standard deviation (SD), median (interquartile range, IQR), or frequency (*n*). MoCA, montreal cognitive assessment; FMA-UE, fugl-meyer assessment upper extremity; ARAT, action research arm test. ^a^Severity categories based on FMA-UE scores: severe (< 33), moderate (33–47), mild (48–66) (37).

Eight professionals evaluated the 18 ARAT-VR items. Most tasks in the Grasp and Gross Movement subscales yielded median difficulty scores of 0 (−1.0–0). However, professionals rated the manipulation of minute objects as more difficult in VR than in the ARAT, specifically the 1 cm alloy tube (1 [0.8–1.0]) and pinching 6 mm marbles (1.0 [1.0–1.0]). Conversely, tasks involving larger objects, such as the 1.5 cm marbles (0 [0–0.5]), were rated as equivalent to the real tasks. The pouring water task was perceived as slightly easier (−0.5 [−1.0–0]). In the technical evaluation, the system received high ratings for tracking stability and ergonomics (Median scores ranged from 4.5 to 5.0), with an overall acceptance score of 5.0 (4.0–5.0). All the scores supporting the data are presented in Supplementary Video 1.

As anticipated in a healthy cohort, a pronounced ceiling effect was observed. According to COSMIN recommendations (defined as >15% of participants achieving the maximum possible score), ceiling effects were present across both assessment modalities. In the traditional ARAT (max 57), 100% (*n* = 30) of participants achieved the maximum score with their dominant hand, and 93.3% (*n* = 28) with their non-dominant hand. Similarly, in the ARAT-VR assessment on Day 1 (max 54), 40.0% (*n* = 12) and 30.0% (*n* = 9) of healthy controls achieved the maximum score for their dominant and non-dominant hands, respectively.

**Figure 4:**
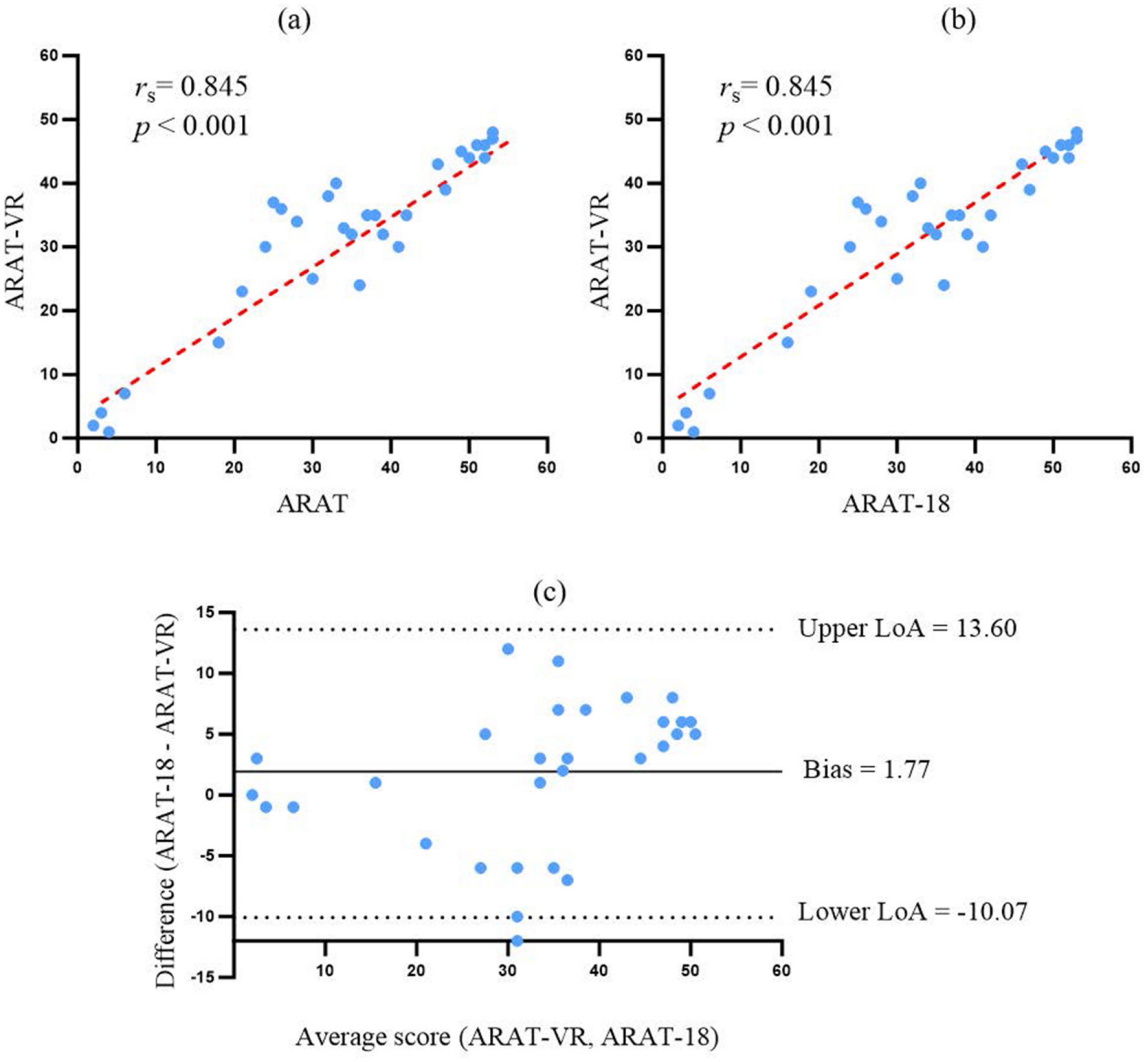
Concurrent validity analysis between ARAT-VR (Day 1) and ARAT scores for the paretic hand in stroke survivors (*N* = 30). **(A)** Scatter plot illustrating the relationship between the ARAT-VR (18 items, max 54) and the adjusted traditional ARAT (18 matched items, max 54). **(B)** Scatter plot comparing the ARAT-VR with the full traditional ARAT (19 items, max 57). **(C)** Bland-Altman plot assessing agreement between the ARAT-VR and the adjusted traditional ARAT (18 items).

Regarding concurrent validity, stroke survivors obtained a median ARAT-VR score on Day 1 of 35.0 (26.3–42.3) and a traditional ARAT-18 score of 35.5 (25.3–46.8) for the paretic hand. A strong positive correlation was found between the ARAT-VR and the ARAT-18 (*r*_s_ = 0.845, 95% CI: 0.673–0.965, *p* < 0.001; Figure 4A), as well as with the full 19-item ARAT (*r*_s_ = 0.845, 95% CI: 0.696–0.961, *p* < 0.001; Figure 4B). Confidence intervals were derived using bias-corrected and accelerated (BCa) bootstrapping with 1,000 resamples. The Wilcoxon signed-rank test revealed no statistically significant difference between the ARAT-18 and ARAT-VR scores (*Z* = −1.582, *p* = 0.114). Bland-Altman analysis (Figure 4C), based on the 18 shared items, revealed a mean bias of 1.77 points, with 95% limits of agreement ranging from −10.07 to 13.60. For the less-affected hand, median scores were 50.0 (49.0–50.8) (ARAT-VR) and 54.0 (53.0–54.0) (ARAT-18).

In terms of performance in healthy controls, subjects achieved median ARAT-VR scores on Day 1 of 53.0 (53.0–54.0) for the dominant hand and 53.0 (52.0–54.0) for the non-dominant hand, compared to a median of 54.0 (54.0–54.0) on the ARAT-18. Spearman correlation analysis showed significant positive correlations between age and the score difference (ARAT-18 minus ARAT-VR Day 1) for both the dominant (*r*_s_ = 0.66, *p* < 0.001) and non-dominant hands (*r*_s_ = 0.57, *p* = 0.001).

No adverse events—such as cybersickness, dizziness, nausea, visual discomfort, or excessive fatigue—were reported by either stroke survivors or healthy controls during or after the ARAT-VR sessions. With respect to usability, the median SUS scores were 85.0 (80.0–89.4) for HCS and 81.3 (75.0–89.4) for stroke survivors. The Mann–Whitney *U*-test indicated no statistically significant difference in total scores between the two groups (*U* = 366, *p* = 0.212). Discriminant validity was confirmed by a Mann–Whitney *U*-test, which showed that ARAT-VR scores for the paretic hand of stroke survivors (Median = 35.0) were significantly lower than those for the non-dominant hand of healthy controls (Median = 53.0; *Z* = −6.69, *p* < 0.001).

Finally, for test-retest reliability (Table 2), the paretic hand demonstrated an ICC of 0.98 (95% CI: 0.963–0.991, *p* < 0.001), and the Wilcoxon test showed no significant difference between Day 1 and Day 7 (*p* = 0.301). The standard error of measurement (SEM) was calculated to be 1.94, with a minimum detectable change (MDC) of 5.36 points. Similarly, for the less-affected hand and healthy controls, Wilcoxon tests revealed no statistically significant difference between sessions (all *p* > 0.05).

**Table 2:** Test-retest reliability of the ARAT-VR across participant groups.

Group/Hand	D1 (Median)	D7 (Median)	ICC (95% CI)	*p*-value (ICC)	*p*-value (Wilcoxon)
Stroke survivors					
Paretic hand	35.0 (26.3–42.3)	35.5 (28.3–42.5)	0.98 (0.963–0.991)	< 0.001	0.301
Less-affected hand	50.0 (49.0–50.8)	51.0 (50.0–51.0)	0.65 (0.382–0.814)	< 0.001	0.072
Healthy controls					
Dominant hand	53.0 (53.0–54.0)	53.0 (53.0–54.0)	0.76 (0.557–0.879)	< 0.001	0.564
Non-dominant hand	53.0 (52.0–54.0)	53.0 (52.3–54.0)	0.80 (0.626–0.900)	< 0.001	0.197

ICC, intraclass correlation coefficient.

## Discussion

To the best of our knowledge, this study provides the first validation of an 18-item ARAT-VR system utilizing optical hand-tracking technology. Building upon previous feasibility studies that adapted subsets of the ARAT (29), the current work extended the digital assessment to include tasks involving complex object geometries and fine motor dexterity. The results indicated strong concurrent correlation but limited absolute agreement with the traditional scale, as well as excellent test-retest reliability. Regarding user acceptance, stroke survivors rated the system's usability favorably, with scores comparable to healthy controls.

To contextualize these findings, we compared our study characteristics and psychometric properties with a previous immersive ARAT-VR validation study by Burton et al. (29) (Table 3). Both systems demonstrated comparable concurrent validity (*r*_s_ > 0.84) and excellent test-retest reliability (ICC ≥ 0.98) for the paretic hand. However, several technological and methodological distinctions are noteworthy. Technologically, while earlier adaptations excluded certain minute objects due to the hardware constraints of the time, the integration of iToF depth sensing in the current setup facilitated the inclusion of 18 items, encompassing fine pinch tasks. Methodologically, to build upon prior research, we utilized consecutive sampling and assessor blinding to further minimize potential selection and observer biases. Additionally, our cohort included a broader distribution of motor impairment severities (from mild to severe), which may help extend the generalizability of the ARAT-VR across different phases of stroke recovery.

**Table 3:** Comparison of study characteristics and psychometric properties with a previous ARAT-VR validation study.

Feature	Current study	Burton et al. (29)
VR Technology	PICO 4 Ultra (iToF depth sensor + 32MP RGB)	Oculus Quest 2 (Standard optical tracking)
Assessment scope	18 Items (Retained complex grasp and fine pinch)	13 Items (Excluded minute objects)
Participant *N*	30 Stroke, 30 Healthy Controls	30 Stroke, 25 Healthy Controls
Sampling approach	Consecutive sampling	Convenience sampling
Blinding procedure	Assessor blinded (Independent raters for VR/Physical)	No blinding (Same assessor for both)
Stroke severity	FMA-UE Mean: 45.4 ± 16.1 (Mild to severe)	ARAT Median: 48.5 (IQR: 20.5–54) (Mildly impaired)
Concurrent validity	*r_*s*_* = 0.845 (ARAT-18 vs. Physical)	*r* = 0.84 (ARAT-13 vs. Physical)
Test-retest ICC	0.98 (95% CI: 0.963–0.991)	0.99 (95% CI not reported)

The strong positive correlation observed between the ARAT-VR and the traditional scale suggests that the virtual assessment successfully captures comparable kinematic and visuomotor control dimensions of upper limb motor function, closely mirroring the spatial trajectories required by the physical reference standard. Furthermore, comparisons between the traditional ARAT-18 and ARAT-VR scores revealed no statistically significant difference, although a trend toward lower scores in the VR modality was observed. Visual inspection of the Bland-Altman plot indicated a uniform distribution of differences across the range of average scores, suggesting that the measurement error of the ARAT-VR is consistent regardless of the stroke survivor's functional ability level. However, a relatively wide 95% limits of agreement (LoA: −10.07 to 13.60) were observed. Importantly, the observed limits of agreement encompass a range wider than the established Minimal Clinically Important Difference (MCID) of the ARAT (estimated at 5.7 points for stroke populations) (38). This suggests that caution is advised when interpreting absolute ARAT-VR scores as direct 1:1 substitutes for conventional scores in a cross-sectional setting. Furthermore, the absence of tactile feedback (TF) in the virtual environment may have contributed to the observed score discrepancies, as patients had to rely entirely on visual cues to gauge grip aperture. (39, 40). Fundamentally, the absence of TF alters the sensorimotor loop. In physical tasks, mechanoreceptors provide critical error correction signals for grasp force modulation (41, 42). In our VR environment, stroke survivors must rely almost exclusively on visual feedback to modulate hand aperture. This sensory reweighting significantly increases the cognitive load and motor difficulty for fine pinch tasks (43). However, it is important to acknowledge that the current study did not directly investigate this mechanism, and this explanation remains speculative. The observed discrepancies may also stem from alternative factors inherent to the virtual environment. For instance, subtle challenges in virtual depth perception, minor kinematic estimations inherent in optical hand-tracking algorithms (44), or the lack of physical object solidity (which permits different grasping strategies than in the real world) could all contribute to score variances (45). Further research may address this issue through optimization, such as incorporating appropriate haptic gloves or integrating corresponding vibration feedback components at the wrist (46, 47).

Consistent with COSMIN guidelines, 100% of healthy controls achieved maximum scores in the physical ARAT, as expected for an impairment scale. Interestingly, this ceiling effect was markedly reduced in the ARAT-VR (40.0%). This discrepancy reflects the technological demands of the virtual environment rather than motor impairment. Lacking tactile feedback, users rely exclusively on visuomotor control and operate more cautiously to ensure accuracy. This directly impacts the temporal-based ARAT-VR scoring, indicating that the VR adaptation introduces universal operational friction, serving as a more stringent test of pure kinematic and visuomotor precision.

Despite this, the ARAT-VR demonstrated excellent test-retest reliability for the paretic hand (ICC = 0.98). The absence of a statistically significant difference between sessions suggests high temporal stability. The SEM was calculated to be 1.94, resulting in an MDC of 5.36 points. This provides a threshold for distinguishing genuine performance changes from random measurement error in future longitudinal studies (48). Furthermore, the primary value of the healthy control data lies not in the ICC—which was inherently constrained by ceiling effects—but in demonstrating the system's specificity: the consistent near-maximal scores across sessions confirm that the ARAT-VR does not produce false positives for motor impairment in unimpaired individuals.

Consistent with previous findings (29, 49), older age was correlated with larger score discrepancies in healthy controls. However, it is important to note that the absolute VR scores remained high across all age groups (Median > 50). This suggests that while age is a factor in initial performance, it does not preclude effective use of the system.

Both stroke survivors and healthy controls rated the usability of the ARAT-VR as “excellent” (Median SUS > 80). The statistical analysis revealed no significant difference in scores between the two groups. This indicates that motor impairments did not hinder the stroke survivors' ability to operate the system effectively. The comparable ratings support the feasibility of implementing the system in rehabilitation settings, as high user acceptance is a prerequisite for patient adherence (50).

The findings of this study support the potential clinical utility of the ARAT-VR as a valid, reliable, and prop-free assessment tool. By automating temporal scoring and reducing the reliance on physical equipment, the system presents a promising supplementary digital tool for standardized evaluation. Furthermore, the system's optical tracking capabilities allow for the passive capture of kinematic data. If further validated, these objective metrics could complement traditional ordinal scores, providing clinicians with supplementary information regarding spatial trajectories to inform the evaluation of movement quality (51, 52).

While the current study focused on psychometric validation within a controlled setting, these results suggest several avenues for future investigation. Logistically, the ARAT-VR could facilitate remote monitoring and home-based functional evaluations via consumer VR hardware (7, 53, 54). However, transitioning to unsupervised home use entails challenges, including mitigating implementation costs, designing intuitive interfaces for older adults, and ensuring robust technical support (55, 56). Additionally, accumulating digital kinematic data may offer opportunities for machine-learning integration to predict recovery trajectories (57, 58). Ultimately, the broader clinical vision is to seamlessly integrate this automated evaluation module with active VR therapeutic interventions. The immersive and interactive nature of VR could alleviate assessment fatigue and enhance patient engagement, thereby facilitating a continuous ‘assessment-training-reassessment' closed-loop ecosystem. Translating these promising technological possibilities into routine care, however, will require dedicated efficacy and implementation trials.

### Limitations

Several limitations should be acknowledged. First, the single-center design, modest sample size, and limited representation of severe stroke profiles restrict external validation. Moreover, lacking global deficit scores (e.g., NIHSS, mRS) or dedicated spasticity assessments (e.g., Modified Ashworth Scale) restricts comprehensive clinical phenotyping. Additionally, variables such as age, cognition, stroke type, and disease duration are potential confounders. Although previous literature suggests these factors may influence VR performance and upper-limb recovery, our sample was insufficient for subgroup analyses. As these clinical covariates significantly influence VR motor performance (59, 60), larger multi-center trials are required. Second, inherent inability to blind participants introduces potential performance biases. Third, the ARAT-VR lacks tactile feedback and physical weight simulation, evaluating visuomotor control rather than force modulation. This sensory gap, alongside the excluded “hand to back of head” item and the absence of external benchmarking for hand-tracking accuracy, alters task fidelity. Additionally, pronounced ceiling effects among healthy controls limit discriminative ability at high functional levels. Furthermore, omitting standardized questionnaires (e.g., SSQ, VRSQ) limits the formal quantitative evaluation of cybersickness, though none were reported. Finally, this validation does not establish the ARAT-VR's superiority over the conventional ARAT; it merely supports its feasibility as a complementary digital tool, not a replacement in routine care.

## Conclusion

The present study demonstrates that the 18-item ARAT-VR, utilizing controller-free optical tracking, possesses strong concurrent correlation with the conventional ARAT and excellent test-retest reliability. These findings support the ARAT-VR as a promising and potentially useful digital assessment tool for evaluating upper-limb motor function in stroke survivors. However, it is imperative to note that the current evidence does not establish the system's superiority over the conventional ARAT, nor does it confirm its immediate readiness for routine clinical implementation. Future large-scale, well-powered studies are required to rigorously evaluate its effectiveness in unsupervised home-based settings and its potential utility as an active rehabilitation intervention.

## Data Availability

The original contributions presented in the study are included in the article/Supplementary material, further inquiries can be directed to the corresponding author/s.
